# Supplementary figures and images for: Relative Telomere Length and Cardiovascular Risk Factors
Source: Biomolecules. 2019 May 17;9(5):192. doi: 10.3390/biom9050192 (PMC6572569; doi:10.3390/biom9050192)

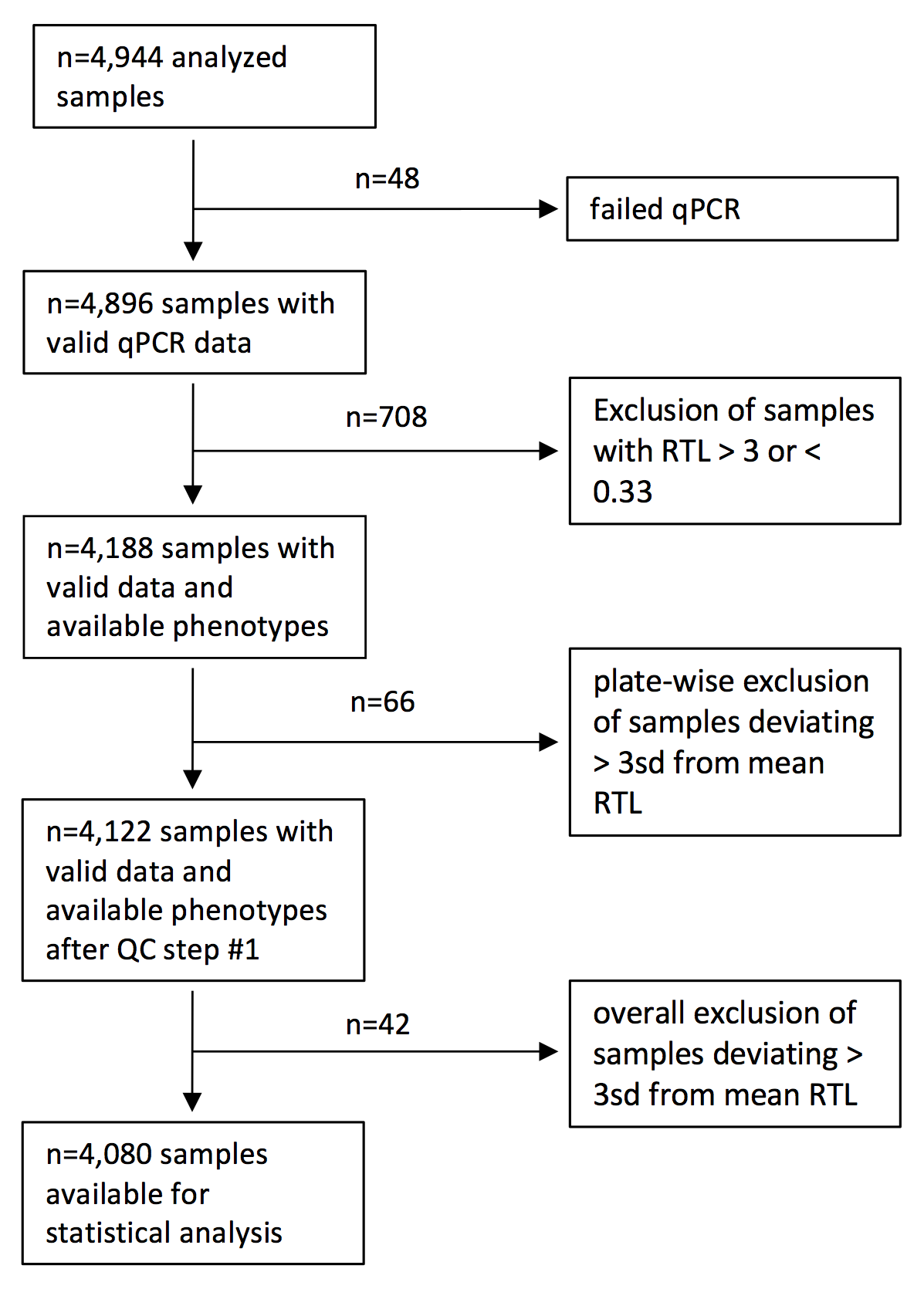

Supplement: Supplementary file 1 [file biomolecules-09-00192-s001.zip › biomolecules-490301-supplementary.tiff]
